# Hemiarthroplasty as management for solitary metastasis of the proximal humerus: a retrospective study

**DOI:** 10.1080/07853890.2026.2719947

**Published:** 2026-08-20

**Authors:** Peng Liu, Shu Lin, Jiang Hu, Jiabing Li, Jiahong Li, Hong Lei, Ji Zhou

**Affiliations:** a Department of Orthopedic Surgery, Sichuan Provincial People’s Hospital, University of Electronic Science and Technology of China, Chengdu, China; b Department of Emergency, Sichuan Provincial People’s Hospital, University of Electronic Science and Technology of China, Chengdu, China

**Keywords:** Proximal humeral metastases, older patients, shoulder arthroplasty, survival analysis

## Abstract

**Purpose:**

Advances in cancer treatment have prolonged survival, but the incidence of bone metastases has also increased. Shoulder arthroplasty for proximal humeral metastases involves major surgical trauma and uncertain functional outcomes in older patients. This study aimed to assess whether age influences prognosis and functional recovery after shoulder arthroplasty.

**Methods:**

Fifty-three patients with isolated proximal humeral metastases undergoing arthroplasty were retrospectively reviewed. Patients were divided into Group A (≥65 years, *n* = 25) and Group B (<65 years, *n* = 28). Surgical outcomes were compared between groups, including operative time, blood loss, hospital stay, pain, limb function, complications, survival, recurrence, and quality of life. Statistical analyses included ANOVA, chi-square tests, and Cox regression to identify prognostic factors.

**Results:**

Operative time, intraoperative blood loss, hospital stay, Visual Analogue Scale, Musculoskeletal Tumor Society scores, complication rates, survival, recurrence, and metastasis showed no significant group differences (*p* > 0.05). However, American Shoulder and Elbow Surgeons scores were significantly different (*p* < 0.05). Cox regression identified tumor malignancy and hospital stay as independent risk factors (*p* < 0.05), while age was not prognostic (*p* > 0.05).

**Conclusions:**

Older patients achieved survival, functional recovery, and complication rates comparable to younger patients. Prosthetic humeral head replacement is therefore a viable treatment option for isolated proximal humeral metastases in older patients.

## 1. Introduction

With the continuous advancement of medical technologies and systemic cancer therapies, the life expectancy of patients with primary malignancies has significantly improved. As a consequence, the incidence of bone metastases has also increased. Among long bones, the humerus is the second most common site for metastatic involvement, following the femur. In particular, the proximal humerus is the most frequently affected site for both primary and secondary tumors of the upper extremity [1,2]. Surgical management has become a critical treatment option for metastatic lesions of the proximal humerus, aiming primarily at pain relief and functional restoration. Advances in surgical techniques and the development of modular prostheses have expanded the options for reconstruction following tumor resection.

Several reconstruction methods have been proposed for managing proximal humeral metastases, including endoprosthetic replacement (EPR), osteoarticular allografts, vascularized bone grafts, and allograft–prosthetic composites (APCs). In cases with pathological fractures, open reduction and internal fixation (ORIF) combined with bone cement augmentation may also be used [3–5]. While bone grafting enables joint surface reconstruction, it is associated with poor healing, early resorption, and complications such as fixation failure or implant migration [6]. ORIF with cement offers immediate stability and minimal invasiveness but may result in inadequate tumor clearance and poor bone healing due to factors such as osteolytic activity and adjuvant therapies [7,8]. In contrast, EPR avoids problems related to bone healing and allows for more radical tumor resection with the formation of tumor-free margins, making it increasingly favored in recent years for proximal humeral lesions [9,10].

However, it remains unclear whether EPR offers the same benefits in older patients, who often have compromised physiologic reserves and higher susceptibility to complications. Questions persist regarding whether advanced age affects survival, recurrence, postoperative pain relief, and functional recovery after shoulder arthroplasty for metastatic bone disease [11]. To address this gap, 53 adult patients with proximal humeral metastases receiving EPR were retrospectively reviewed and stratified by age to evaluate the influence of age on surgical outcomes and prognosis.

## 2. Materials and methods

### 2.1. Patient information

A retrospective review was conducted on patients who underwent proximal humeral hemiarthroplasty (PHH) for solitary metastatic lesions of the proximal humerus at our institution between 2013 and 2021. All patients underwent wide tumor excision using Malawer type I resection. This retrospective cohort study was reported in accordance with the Strengthening the Reporting of Observational Studies in Epidemiology (STROBE) guidelines for cohort studies [12]. This study was approved by the institutional ethics committee of our hospital and conducted under the guidance of the Declaration of Helsinki.

### 2.2. Surgical indications and preoperative preparation

The surgical indications included pathological fractures, severe pain, functional impairment of the shoulder joint, and resistance to radiotherapy. The primary tumor site was identified through clinical history, imaging studies, or pathological biopsy. Inclusion criteria required the absence of other detectable metastatic lesions, favorable prognostic characteristics, and an estimated life expectancy of more than three months.

All patients were preoperatively diagnosed with solitary metastatic lesions by either core needle biopsy or imaging evaluation. The extent of the tumor in the shoulder region was assessed using both computed tomography (CT) and magnetic resonance imaging (MRI). Whole-body PET-CT was adopted as the primary imaging examination to define solitary metastasis and screen systemic distant metastases; patients were only categorized as having solitary proximal humeral metastasis when no other hypermetabolic metastatic lesions were detected throughout the body on PET-CT. All patients underwent proximal humeral hemiarthroplasty, and tumor-specific prostheses were selected based on the extent of bone resection required.

### 2.3. Inclusion and exclusion criteria

Inclusion criteria were as follows: (i) presence of a solitary proximal humeral metastasis confirmed by imaging or biopsy, (ii) pathological fracture, or severe osteolytic lesion with impending fracture (Mirels score ≥9), (iii) poor response to radiotherapy based on RECIST 1.1 criteria, (iv) persistent severe shoulder pain or significant loss of shoulder function, and (v) estimated life expectancy of more than 3 months. Exclusion criteria included: (i) multiple bone metastases (≥2 skeletal sites), (ii) presence of metastatic lesions involving the scapula or axillary nerve/plexus, (iii) death during the perioperative period, (iv) presence of three or more significant comorbidities (e.g. uncontrolled diabetes, severe cardiopulmonary disease, or chronic renal failure), and (v) patients lost to follow-up within 3 months postoperatively. A total of 68 patients with proximal humeral metastases who received surgical treatment at our center from 2013 to 2021 were initially screened *via* institutional electronic medical records. Fifteen patients were excluded according to the above exclusion criteria: 7 with multiple bone metastases, 3 with scapula or axillary nerve plexus invasion, 2 with early loss to follow-up, 2 with three or more severe uncontrolled comorbidities, and 1 perioperative death. The remaining 53 eligible patients formed the study cohort, stratified into Group A (≥65 years, *n* = 25) and Group B (<65 years, *n* = 28) for comparative analysis.

### 2.4. Surgery producer

Surgical incisions were designed according to the location of pathological fractures, extent of soft tissue invasion, and biopsy tract. En bloc wide resection of the tumor-bearing bone was performed with strict adherence to tumor-free principles. The axillary nerve and deltoid muscle were preserved in all patients; no patient required resection of deltoid muscle or axillary nerve during tumor resection. The axillary nerve and deltoid muscle were preserved whenever possible. The osteotomy was made 2–3 cm distal to the tumor margin. Tumor-specific prostheses were selected based on the length of resection, and all prosthetic stems were fixed with bone cement. After prosthesis implantation, surrounding soft tissues were reconstructed. Ligament Advanced Reinforcement System (LARS) was selectively applied only in patients with severe shoulder joint capsule and soft tissue defects after tumor resection to reconstruct the joint capsule and reinforce shoulder stability; LARS augmentation was not required for cases with intact native periarticular soft tissues.

### 2.5. Adjuvant and therapy

Postoperatively, patients began passive and active-assisted movements of the elbow, wrist, and hand early after surgery. A sling was used for immobilization for six weeks. From week 6 onward, passive and assisted active exercises of the shoulder were initiated, with gradual progression to active use as tolerated after 12 weeks. To minimize interference with wound healing, all patients received chemotherapy and/or radiotherapy starting three months after surgery. Chemotherapy and biological therapy were administered for patients with lung, liver, colon, rectal, and gastric cancers. Breast and prostate cancer patients received adjuvant chemotherapy and hormonal therapy, respectively. Radiotherapy was applied in patients with thyroid carcinoma.

### 2.6. Follow-up

Patients were followed up every three months until death, and data related to tumor recurrence and quality of life were collected. Pain was assessed using the Visual Analogue Scale (VAS), while functional outcomes were evaluated using the Musculoskeletal Tumor Society (MSTS) scoring system and the American Shoulder and Elbow Surgeons (ASES) score [13,14]. Local recurrence was monitored *via* X-ray and CT scans, and any suspicious findings were further confirmed by MRI.

### 2.7. Statistical analysis

Kaplan–Meier survival analysis was performed to compare overall survival between the groups, and mean survival times were calculated accordingly. All data are presented as mean ± standard deviation (SD). Differences in means between groups were evaluated using one-way analysis of variance (ANOVA) or the chi-square test, as appropriate. A Cox proportional hazards regression model was used to assess the impact of age on postoperative survival outcomes. All statistical analyses were performed using SPSS software, version 16.0 (SPSS Inc., Woking, United Kingdom), and a p-value of <0.05 was considered statistically significant.

## 3. Results

### 3.1. Patient demographics and surgical characteristics

Fifty-three patients (25 males, 28 females) with histologically confirmed solitary proximal humeral metastatic lesions were enrolled, aged 43–82 years. Subjects were stratified into Group A (≥65 years, *n* = 25) and Group B (<65 years, *n* = 28). All lesions were unilateral and limited to the proximal one-third of the humerus without scapular invasion. Primary tumor origins are listed in Table 1: lung (*n* = 11), breast (*n* = 15), prostate (*n* = 5), thyroid (*n* = 5), colorectal (*n* = 4; colon *n* = 2, rectal *n* = 2), renal cell carcinoma (*n* = 9), hepatocellular carcinoma (*n* = 1), gastric (*n* = 1), uterine (*n* = 1), and unknown primary (*n* = 1). Twenty-two patients had prior primary tumor resection, 19 had chronic comorbidities (diabetes, hypertension, chronic gastritis, pulmonary emphysema), and 19 presented with pathological fractures. The mean age was 72.44 ± 4.97 years in Group A and 54.75 ± 4.48 years in Group B, with a significant intergroup difference (*p* = 0.000). No between-group differences were detected in gender distribution (*p* = 0.909), primary tumor type (*p* = 0.921), tumor malignancy grade (*p* = 0.926), or pathological fracture rate (*p* = 0.983) (Table 1). Surgical parameters including operative time, intraoperative blood loss, transfusion volume, and hospital stay were comparable between the two groups (all *p* > 0.05, Table 2).

**Table 1.** Demographic data for patients with proximal humerus metastases.

Variable	Group A (*n* = 25)	Group B (*n* = 28)	χ^2^/*F*	*p*
Gender (male/female)	12/13	13/15	0.013	0.909
Age (years, mean ± SD)	72.44 ± 4.97	54.75 ± 4.48	185.978	0.000
Mean body mass index (kg/m^2^)	21.16 ± 2.25	22.25 ± 1.94	3.595	0.064
Basic illnesses (Yes/No)	11/14	8/20	1.367	0.242
Resection of the primary tumor (Yes/No)	9/16	13/15	0.592	0.442
Pathological fracture (Yes/No)	9/16	10/18	0.000	0.983
Primary malignancy				
Lung	5	6	4.513	0.921
Liver	0	1		
Kidney	4	5		
Prostate	3	2		
Breast	7	8		
Thyroid	2	3		
Colon	1	1		
Rectal	1	1		
Gastric	0	1		
Uterus	1	0		
Unidentified	1	0		
Degree of malignancy				
High	6	8	0.153	0.926
Moderate	6	6		
Low	13	14		

Data are presented as mean ± standard deviation or number (percentage). χ², Chi-square test; *F*, one-way analysis of variance (ANOVA). *p* < 0.05 indicates statistical significance.

**Table 2.** Surgery, hospitalization and prognosis data of patients with proximal humerus metastases.

Variable	Group A (*n* = 25)	Group B (*n* = 28)	*F*	*p*
Surgery and hospitalization (mean ± SD)				
Surgery duration (min)	211.63 ± 65.78	209.86 ± 65.17	0.010	0.922
Resection length(cm)	12.43 ± 2.80	11.97 ± 2.37	0.412	0.524
Blood loss (ml)	660.00 ± 294.85.29	762.50 ± 273.06	1.660	0.203
Blood transfusion (ml)	376.00 ± 284.72	435.71 ± 271.09	0.611	0.438
Hospitalized time (day)	18.72 ± 2.59	18.61 ± 5.17	0.010	0.922
Improvement after surgery (mean ± SD)				
VAS	4.52 ± 1.39	5.25 ± 1.97	2.369	0.130
MSTS (%)	17.52 ± 3.02	17.18 ± 3.78	0.130	0.720
ASES	15.48 ± 2.35	14.29 ± 1.86	4.252	0.044*

VAS, Visual Analogue Scale; MSTS, Musculoskeletal Tumor Society score; ASES, American Shoulder and Elbow Surgeons score. *Statistically significant difference (*p* < 0.05).

### 3.2. Pain, function, and quality of life

The follow-up duration ranged from 4 to 52 months in Group A (mean: 19.80 ± 11.18 months) and from 3 to 51 months in Group B (mean: 19.36 ± 11.66 months), with no significant difference between the two groups (*p* = 0.889). Postoperatively, the mean reduction in VAS pain score was 4.52 ± 1.39 in Group A and 5.25 ± 1.97 in Group B, showing no statistically significant difference (*p* = 0.130). All 53 patients exhibited varying degrees of upper limb dysfunction before surgery. Following surgery, the mean increase in MSTS score was 17.52 ± 3.02 in Group A and 17.18 ± 3.78 in Group B, with no significant difference (*p* = 0.720). Similarly, improvements were observed in ASES scores in both groups, with an average increase of 15.48 ± 2.35 in Group A and 14.29 ± 1.86 in Group B, with a statistically significant difference (*p* = 0.044) (Table 2).

### 3.3. Survival status and cox regression

**Figure 1.**
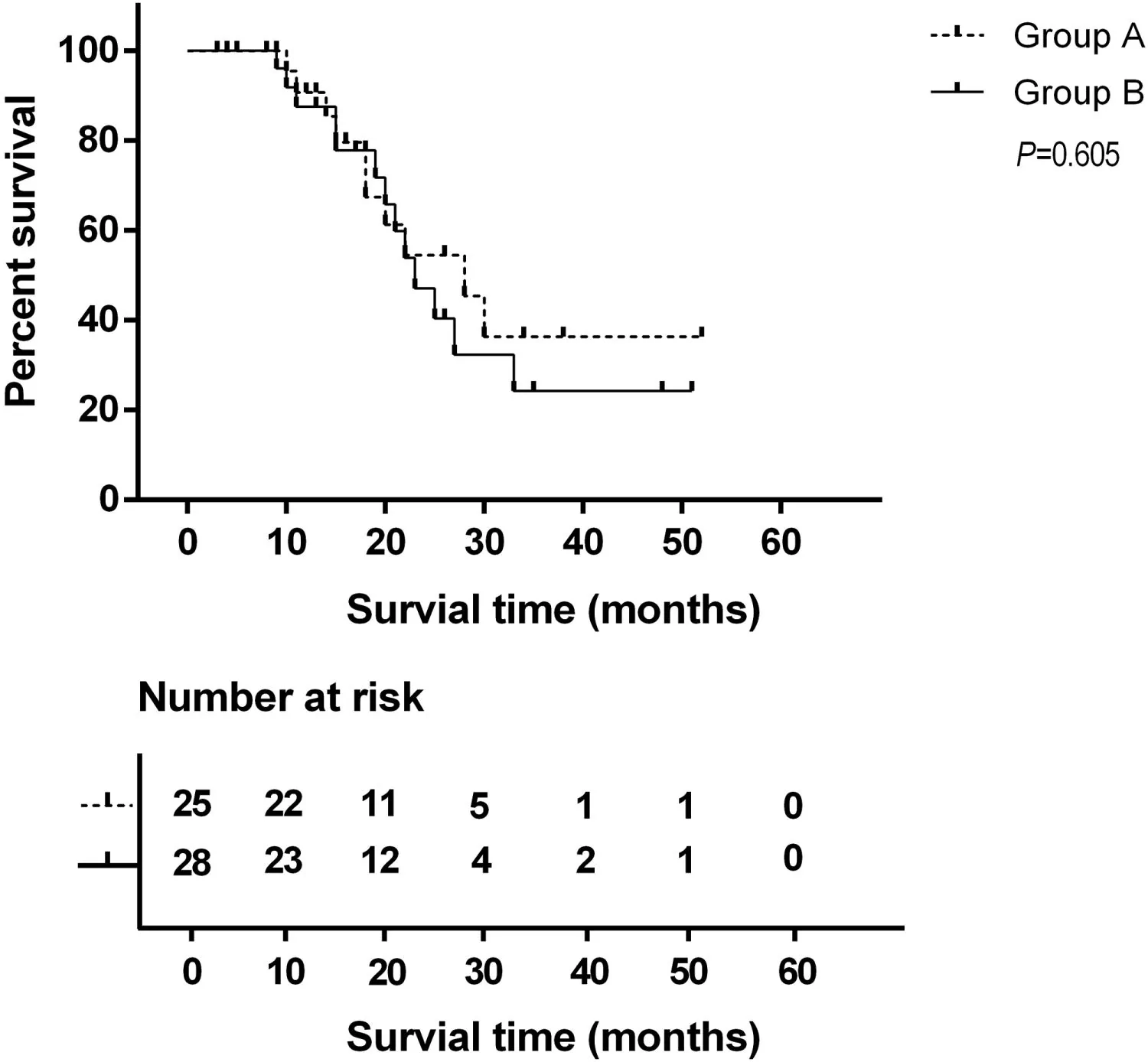
The survival curves after surgery of two groups: older group (≥65 years old, *n* = 25) and young group (<65 years old, *n* = 28).

In Group A, two patients died within the first-year post-surgery. The 1-year survival rate was 90.7% ± 6.3%, and the 2-year survival rate was 54.5% ± 12.2%. In Group B, three patients died within the first-year post-surgery. The 1-year survival rate was 87.5% ± 6.8%, and the 2-year survival rate was 47.1% ± 12.0%. The median survival time in Group A was 28.0 ± 5.3 months [95% confidence interval (CI): 17.7–38.3 months], while in Group B, it was 23.0 ± 2.5 months (95% CI: 18.2–27.8 months). The mean survival time in Group A was 31.7 ± 4.2 months (95% CI: 23.5–39.8 months), and in Group B, it was 28.0 ± 3.5 months (95% CI: 21.2–34.9 months). Log-rank test indicated no significant difference between the two groups (*p* = 0.605) (Figure 1). Subgroup survival analysis stratified by prior primary tumor resection was additionally performed. Log-rank test showed no significant difference in overall survival between patients with and without preoperative primary tumor excision (*p* = 0.945). Furthermore, a Cox proportional hazards regression model was constructed including age, tumor malignancy grade, length of hospital stays, resection extent, complications and prior primary tumor resection as covariates to identify independent predictors of overall survival. The analysis revealed that age was not a significant risk factor for mortality (*p* = 0.416). However, tumor characteristics and hospitalization were found to be significant predictors of postoperative survival (*p* = 0.017; *p* = 0.040) (Table 3).

**Table 3.** Results of the cox regression model analysis.

Covariate	Regression coefficient	Standard error	*Wald*	*p*	Relative risk
Age	−0.019	0.024	0.662	0.416	0.981
Character of tumor	0.730	0.307	5.667	0.017*	2.074
Hospitalization	0.105	0.051	4.210	0.040*	1.111
Resection size	0.130	0.110	1.397	0.237	1.139
Complications	−1.053	0.645	2.668	0.102	0.349
Primary tumor excision	−0.151	0.481	0.098	0.754	0.860

*Statistically significant difference (*p* < 0.05). Global model χ² = 19.149, *p* = 0.004.

### 3.4. Local recurrence and metastasis

No local recurrence was observed in either group within follow up. In Group A, 5 out of 25 patients (20.00%) developed metastases in other locations, while in Group B, 8 out of 28 patients (28.57%) had metastases in other locations. There was no statistically significant difference between the groups (*p* = 0.469). Most metastases occurred within the first 1–2 years, with an average time of 1.4 ± 0.2 years.

### 3.5. Perioperative complications

In Group A, there were 8 perioperative complications (32%), including 4 cases of wound complications (1 of which was an infection), 2 cases of subluxation and 2 cases of prosthesis loosening occurring 1 year postoperatively. In Group B, there were 8 perioperative complications (28.57%), including 3 cases of subluxation, 3 cases of wound complications, and 2 cases of prosthesis loosening occurring 1 year postoperatively. There was no significant difference between the two groups (*p* = 0.786) (Table 4).

**Table 4.** Complication, recurrence and metastasis data of patients with proximal humerus metastases.

Variable	Group A (*n* = 25)	Group B (*n* = 28)	χ^2^	*p*
Complications				
Subluxation	2 (8%)	3 (10.71%)	0.074	0.786
Wound complication	4 (16%)	3 (10.71%)		
Implant loosening	2 (8%)	2 (7.14%)		
Periprosthetic fracture	0 (0%)	0 (0%)		
Local recurrence	0 (0%)	0 (0%)	/	/
Metastasis	5 (20%)	8 (28.57%)	0.524	0.469

Data are presented as number (percentage). χ², Chi-square test. *p* < 0.05 indicates statistical significance.

## 4. Discussion

### 4.1. Considerations for surgical approach in older patients

A relatively strict set of inclusion criteria was adopted given the cautious strategy for joint replacement among elderly patients with proximal humeral metastases. Accordingly, surgery was performed only for elderly patients presenting with pathological fractures, severe pain combined with substantial functional impairment, or impending fractures [15]. Patients in Group A generally showed more severe preoperative pain and functional limitations relative to Group B. To exclude the potential confounding effect of these baseline discrepancies on study endpoints, changes in preoperative and postoperative VAS, MSTS, and ASES scores were selected as the primary evaluation indicators.

### 4.2. Surgical strategy selection in older patients

Shoulder arthroplasty includes hemiarthroplasty (humeral head replacement) and reverse shoulder arthroplasty. For older patients with metastatic lesions of the proximal humerus, surgical decision-making must balance tumor control, postoperative functional recovery, and patient tolerance [16,17]. Cemented hemiarthroplasty eliminates the need for bone healing, reducing infection risk and making it ideal for patients with limited life expectancy or those needing early mobilization [18,19]. Although reverse shoulder arthroplasty may achieve better postoperative function in some patients, it involves a more extensive surgical procedure, and in oncologic patients, the potential risk of tumor progression should be carefully weighed. Accordingly, cemented hemiarthroplasty with or without LARS reconstruction was adopted for all patients in this cohort.

### 4.3. Pain relief outcomes

Pain relief is the primary goal of surgery. The rate of complete pain relief after cemented EPR could reach 55%, with no significant difference between age groups, as bone cement provides immediate stability and reduces micromotion-related pain [6]. After intramedullary nailing combined with cement augmentation, both older and younger patients experienced *a* ≥ 4-point reduction in VAS pain scores [20]. This is similar with the pain relief observed in our study following cemented hemiarthroplasty, further supporting the notion that age does not significantly influence analgesic outcomes, and that the effectiveness of pain relief depends more on the completeness of tumor resection and postoperative stability.

### 4.4. Functional demands and clinical value in older patients

Our study also showed that although older patients had slightly reduced shoulder range of motion (e.g. forward flexion and abduction) compared to the younger group, improvements in functional scores such as MSTS (calculated as percentage of the full 30-point raw scale) did not differ significantly. It was reported that postoperative MSTS scores across age groups consistently ranged between 60% and 70% [21]. Consistently with this standard percentage-based reporting format, our cohort achieved a mean relative improvement of 17% in MSTS functional percentage, rather than a 17-point gain in absolute raw score. This may be attributed to the fact that older patients generally have lower functional demands (e.g. hand positioning and basic daily activities), and the ‘spatial positioning function’ provided by the prosthesis is sufficient to meet their needs. Even though younger patients typically possess greater physiological recovery potential, older patients in our study demonstrated better postoperative ASES scores. This may reflect their lower functional expectations, heightened perception of pain relief, and higher subjective satisfaction [22,23]. These findings emphasize that surgical outcomes should be interpreted in the context of patient age, lifestyle, and expectations, rather than relying solely on objective functional metrics. Notably, the axillary nerve and deltoid muscle were preserved in all patients. This eliminates this critical functional confounder and strengthens the reliability of our intergroup functional comparison.

### 4.5. Risk factors affecting long-term survival

Our study identified tumor malignancy as a key risk factor affecting long-term survival in patients undergoing joint replacement for proximal humeral metastases. Higher malignancy was associated with significantly lower survival, with high-risk patients facing a more than twofold greater mortality risk compared to low-risk patients (relative risk = 2.074). This underscores that despite effective local tumor control *via* surgery, the biological aggressiveness of the tumor predominantly determines prognosis, highlighting the importance of integrating tumor biology into surgical decision-making and patient counseling [24,25]. Additionally, our analysis revealed that longer hospital stay was independently linked to prognosis, while recorded postoperative complications were not. This seemingly paradox may be explained by the fact that hospital stay duration reflects a broader range of clinical factors – including physiological frailty, delayed recovery, and unmeasured perioperative challenges – that are not fully captured by complication coding [26,27]. Moreover, prolonged hospitalization may delay systemic treatments essential for metastatic disease control. Thus, length of stay may serve as a more sensitive and integrative indicator of long-term prognosis than the presence of complications alone, but this risk is not high (relative risk = 1.111). Prior primary tumor resection was also incorporated into the Cox regression model, whereas this factor showed no significant association with overall survival in the cohort. A possible explanation is that metastatic lesions already represented systemic tumor burden regardless of whether the primary lesion was removed in advance.

### 4.6. Limitations and future perspectives

This study has several limitations that warrant consideration. First, the sample size is relatively small, which may affect the generalizability of the findings. Given the heterogeneity of metastatic lesions and patient comorbidities in the older population, larger multicenter studies are needed to validate the outcomes of cemented hemiarthroplasty in this specific group. Second, this was a retrospective analysis, and thus may be subject to selection bias and incomplete data. Functional assessments were based on short- to mid-term follow-up, and long-term prosthesis survival, durability of soft tissue reconstruction, and oncologic outcomes were not evaluated. Third, while hemiarthroplasty is recommended as the preferred procedure for elderly patients with proximal humeral metastases, relevant randomized controlled trials that directly compare hemiarthroplasty with reverse total shoulder arthroplasty or other reconstructive methods remain absent. Future prospective trials could clarify optimal indications and develop evidence-based treatment algorithms tailored to life expectancy, tumor burden, and shoulder function.

## 5. Conclusion

This study shows that advanced age is not an independent risk factor for poor prognosis in patients undergoing shoulder arthroplasty for proximal humeral metastases. Instead, tumor aggressiveness and prolonged hospital stay are key predictors of survival. Cemented hemiarthroplasty combined with soft tissue reconstruction effectively addresses the functional needs of older patients, offering reliable pain relief, early mobility, and improved quality of life.

## Data Availability

The datasets generated and/or analyzed during the current study are available from the corresponding author on reasonable request.
